# GHCR—A dataset for Grantha handwritten character recognition

**DOI:** 10.1016/j.dib.2024.110783

**Published:** 2024-08-06

**Authors:** Basaraboyina Yohoshiva, Nagendra Panini Challa

**Affiliations:** VIT-AP University, Amaravati, Andhra Pradesh, India

**Keywords:** Handwritten character recognition, Machine vision, Optical recognition systems, Machine learning, Deep learning

## Abstract

This dataset presents a comprehensive collection of handwritten Grantha characters, comprising numbers and vowels, gathered from participants spanning diverse age groups. Utilizing standard A4 sheets, participants were instructed to handwrite Grantha characters. The Grantha script encompasses 10 numbers and 34 vowels. The Grantha Character dataset comprises 44 distinct characters of numbers and vowels.

A dataset comprising 133 handwritten samples for each number and 133 for each vowel was collected. These samples underwent digitization and preprocessing steps, including segmentation, resizing, and grayscale conversion. The final dataset consists of 5852 images, comprising 1330 samples for numbers and 4522 samples for vowels. The data is provided in both image and CSV formats, accompanied by corresponding labels.

facilitating its utilization in machine learning model development. With limited datasets available for the Grantha script, this contribution addresses a significant gap by providing a benchmark dataset for Grantha numeral and vowel recognition.

Moreover, this novel dataset serves as a fundamental resource for commencing machine learning research in Indian languages that have historical connections to the Grantha script.

Specifications Table

**Table:** 

Subject	Computer Vision and Patter Recognition
Specific subject area	Handwritten Character Recognition, Machine Vision, Optical Recognition Systems, Machine Learning (ML), Deep Learning (DL).
Type of data	Image: JPG Table: comma-separated values (CSV)
Data collection	Participants from colleges, aged between 15 and 55, were requested to handwrite Grantha numbers and vowels on standard A4 sheets Subsequently, these sheets were digitized by scanning them using a smartphone.we used the Direct Observation Method for data collection in our study. Specifically, our data collection process involved the following steps: • Participants were provided with a printed template of the Grantha script, which included both vowels and numbers. • They were then instructed to write these characters on an A4 white plain sheet using a regular pen. By employing the Direct Observation Method, we were able to gather direct data on participants.
Data source location	VIT-AP University, Inavolu beside AP Secretariat, Amravati India
Data accessibility	Repository name: Public Repository for Grantha Data Set Data identification number: 10.17632/j89cdpxwmw.2 Direct URL to data: https://data.mendeley.com/drafts/j89cdpxwmw

## 1 Value of the Data

• Grantha characters were obtained from participants representing a range of age groups. These characters were subsequently preprocessed, resized, and labeled, facilitating the development of ML models.
• As of the current date, datasets in the Grantha script are scarce. This unique dataset offers one of the largest collections of handwritten data in the Grantha script, aiding in the development and refinement of ML models for computer vision [1,8,9].
• Additional researchers can employ this dataset as a reference standard for Handwritten character identification of Grantha numbers and vowels [[2], [3], [4], [5], [6], [7]].
• The dataset comprises 5852 individuals isolated Grantha characters, consisting of 1330 numbers and 4522 vowels. With this significant number of samples, it is highly suitable for model building in DL research.
• The absence of a benchmark dataset, akin to Modified National Institute of Standards and Technology database for Latin numbers, has hindered research on Grantha numerals. Hence, this dataset aims to bridge the existing data gap and foster further research in this domain.
• Numerous Indian languages, such as Tamil, Kannada, Malayalam, Telugu, Tulu, and others, have historical connections to the Grantha Script. Consequently, this novel dataset can serve as a fundamental resource for commencing ML Research in these ancient languages.

## 2 Background

The motivation behind compiling this dataset stems from the significant scarcity of digitized Grantha script data, which is essential for developing robust machine learning (ML) and deep learning (DL) models for script identification and recognition. Grantha script, historically used in South India for writing Sanskrit, has limited contemporary digital resources due to its ancient origin and complex character structure. This dataset was created to bridge this gap by providing a comprehensive collection of handwritten Grantha script samples. The data was meticulously gathered from various subjects, digitized, and converted into a CSV format to facilitate ease of use in ML and DL modeling. By providing a structured and accessible dataset, we aim to foster advancements in computer vision applications and historical document analysis related to Grantha script.

## 3 Data Description

**Fig. 1:**
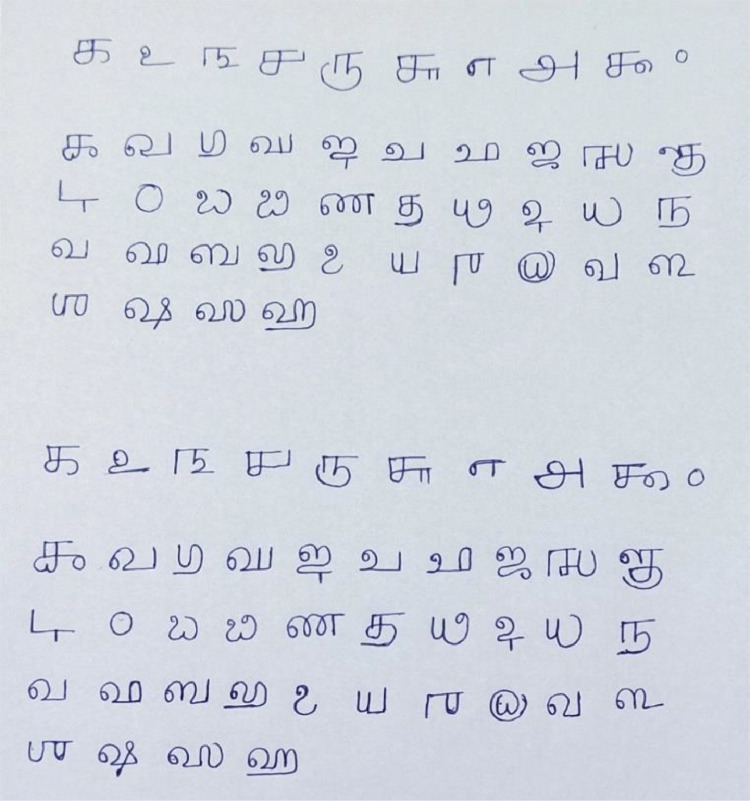
Sample A4 Sheet used to collect data.

The dataset comprises handwritten samples of Grantha numbers and vowels, totalling 44 different characters (10 numbers and 34 vowels), as detailed in Table 1, Table 2. The data collection process involved specific steps. Participants were provided with a printed template of the Grantha script, which included both vowels and numbers. They were then instructed to write these characters on an A4 white plain sheet using a regular pen. Subsequently, the standard A4 sheets containing handwritten characters were scanned using a mobile phone, as depicted in Fig. 1.

**Table 1:** Representation of Grantha Numbers.

**Table 2:** Representation of Grantha vowels.

### 3.1 Basic statistics

Table 3 presents the basic statistics for the collected data, categorized by age group, gender, participant details, handedness, level of expertise, and prior experience with the Grantha script.

**Table 3:** Basic statistics for data.

Statistic	Details
Age Group	15–55 years
Gender	Male and Female
Male Participants	75
Female Participants	75
Handedness	All participants are right-handed
Level of Expertise	Students and staff of VIT-AP University
Prior Experience with Grantha Script	None

### 3.2 Flowchart for data collection

After the initial data collection, additional digitization steps were undertaken. Specifically, the VIVO V17Pro smartphone with a 48 MP back camera was utilized to digitize the hard copies of the handwritten sheets. This process ensured the conversion of physical data into digital format for further analysis and processing. Furthermore, the data underwent segmentation, OpenCV-Python version 4.9.0, a widely-used computer vision library known for its effectiveness in image processing tasks, was employed. This approach facilitated the automatic identification and extraction of characters from the scanned images, enhancing the efficiency and accuracy of the data processing pipeline.

Moreover, the processed data underwent additional preprocessing steps to prepare it for analysis. This included resizing the images and converting them to grayscale, a common practice in image processing to simplify subsequent computations and analyses.

Finally, to ensure data quality and accuracy, a manual review process was implemented. All images underwent manual review to verify proper segmentation and to address any potential errors or inconsistencies. This meticulous review process aimed to enhance the reliability and integrity of the dataset, ensuring its suitability for various research applications.

**Fig. 2:**
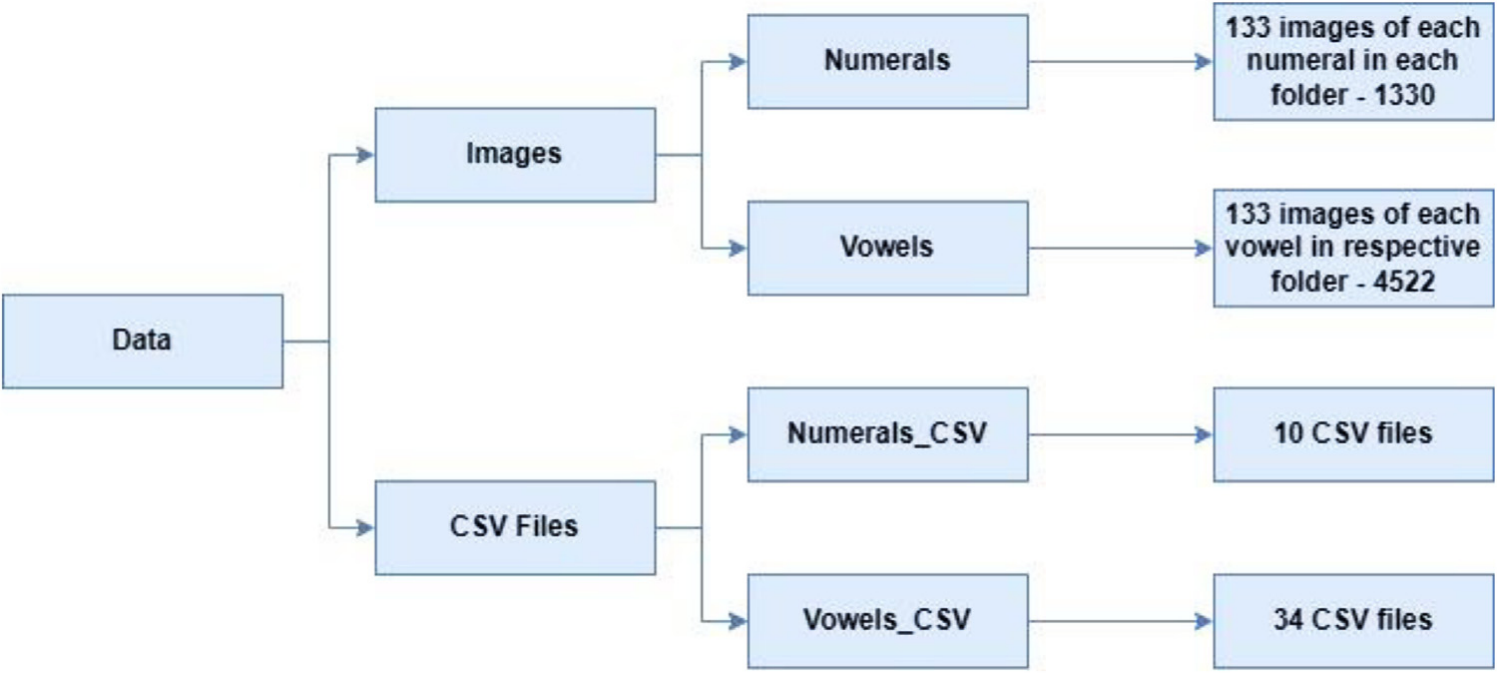
Data arrangement.

The dataset comprises 5852 digitized images, consisting of 1330 Grantha numbers (133 samples for each number) and 4522 vowels (133 sample for each vowel). This data was meticulously organized in distinct folders, as depicted in Fig. 2. The dataset includes a total of 44 CSV (comma separated values) files, with each file representing a unique character or number. Each folder of images aligns with its corresponding category.

## 4 Experimental Design, Materials and Methods

### 4.1 Data gathering

Research into recognizing handwritten English characters has achieved notable success, unlike Indian scripts such as Grantha. While deep learning techniques show proficiency in automatically recognizing Grantha handwritten characters, the availability of benchmark datasets with labels is crucial. This dataset endeavors to address the Data Deficiency for Grantha numbers and vowels.

Participants were directed to inscribe isolated Grantha numbers and vowels on standard A4 sheets, as illustrated in Fig. 1. Data collection encompassed individuals across various age groups, ranging from 15 to 55 years, thus ensuring a diverse range of data samples.

### 4.2 Data handling

All documents were scanned using a smartphone and saved in JPG format. Characters were isolated from the scanned JPG images using the bounding box method, and then carefully segmented to eliminate any noise. Following this, all the images were adjusted to dimensions of 28 × 28 pixels and underwent manual verification. The primary objective of creating this dataset is to enhance ML models.

The extracted images underwent conversion to black and white, with the background set to white and the characters to black, as outlined in Table 1, Table 2. Organized into distinct folders, there are a combined count of 44 directories for each character, as depicted in Fig. 2.

**Fig. 3:**
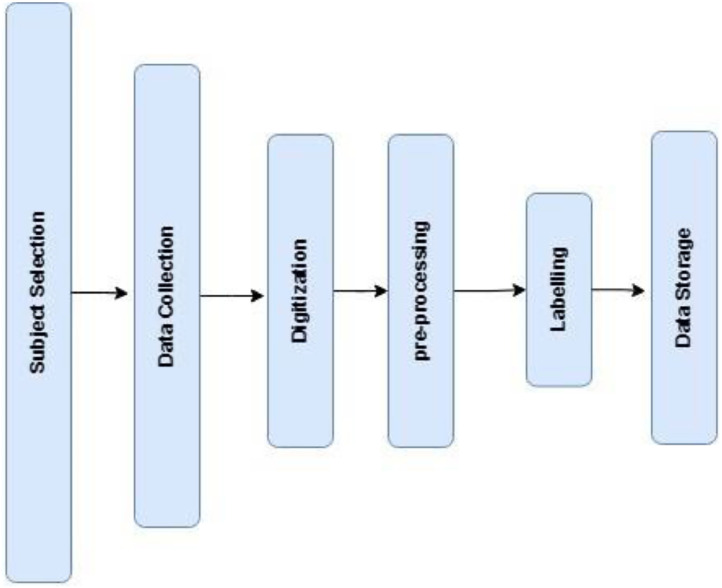
Flowchart for data collection.

Each image was transformed into an image vector, with a corresponding label assigned to it. A 28 × 28 image generates a vector of 1 × 784, with an additional value indicating its label. In total 44 CSV files were created, each representing a distinct character. Each comma separated values (CSV) file contains 133 rows for numerical characters and 133 rows for vowels, where each row corresponds to one image, and the final value denotes the label. The overall data preparation process was depicted in Fig. 3.

## Limitations

First, the data collection was manual, introducing variability in handwriting styles and quality. Although this variability can enhance the robustness of ML models, it also presents challenges in achieving consistent recognition accuracy. Second, the dataset size, though substantial compared to existing resources, may still be limited for training highly sophisticated models that require extensive data.

## Ethics Statement

All handwritten characters were acquired with consent from the respective college authorities prior to data collection. Ethical approval was not deemed necessary as the research did not involve human subjects or animals.

## CRediT authorship contribution statement

**Basaraboyina Yohoshiva:** Conceptualization, Methodology, Writing – original draft. **Nagendra Panini Challa:** Supervision.

## Data Availability

Public Repository for Grantha Data Set (Original data) (Mendeley Data).
